# Health-related quality of life in injury patients: the added value of extending the EQ-5D-3L with a cognitive dimension

**DOI:** 10.1007/s11136-019-02156-2

**Published:** 2019-03-18

**Authors:** Robbin H. Ophuis, Mathieu F. Janssen, Gouke J. Bonsel, Martien J. Panneman, Suzanne Polinder, Juanita A. Haagsma

**Affiliations:** 1 Department of Public Health, Erasmus MC University Medical Center, PO Box 2040, 3000 CA Rotterdam, The Netherlands; 2 Section Medical Psychology and Psychotherapy, Department of Psychiatry, Erasmus MC University Medical Center, Rotterdam, The Netherlands; 3 Division Mother and Child, Utrecht University Medical Center, Utrecht, The Netherlands; 4 Consumer Safety Institute, Amsterdam, The Netherlands

**Keywords:** Health-related quality of life, EQ-5D, Injury, Trauma, Cognition

## Abstract

**Introduction:**

The EQ-5D is frequently used to understand the development of health-related quality of life (HRQL) following injury. However, the lack of a cognition dimension is generally felt as disadvantageous as many injuries involve cognitive effects. We aimed to assess the added value of a cognitive dimension in a cohort of injury patients.

**Methods:**

We analyzed EQ-5D-3L extended with cognition (EQ-5D + C) dimension responses of 5346 adult injury patients. We studied dimension dependency, assessed the additional effect of the cognitive dimension on the EQ-VAS, and, using the EQ-VAS as a dependent variable, determined the impact of EQ-5D and EQ-5D + C attributes in multivariate regression analyses.

**Results:**

Extreme cognitive problems combined with no problems on other dimensions are uncommon, whereas severe problems on other dimensions frequently occur without cognitive problems. The EQ-VAS significantly decreased when cognitive problems emerged. Univariate regression analyses indicated that all EQ-5D + C dimensions were significantly associated with the EQ-VAS. Exploratory analyses showed that using any set of five of the six EQ-5D + C dimensions resulted in almost identical explained variance, and adding the remaining 6th dimension resulted in a similar additional impact.

**Conclusions:**

The addition of the cognition dimension increased the explanatory power of the EQ-5D-3L. Although the increase in explanatory power was relatively small after the cognition dimension was added, the decrease of HRQoL (measured with the EQ-VAS) resulting from cognitive problems was comparable to the decreases resulting from other EQ-5D dimensions.

## Introduction

Due to improved survival rates, more individuals experience long-term consequences of injury. Assessing variations of health-related quality of life (HRQL) following injury is valuable to inform patients and to improve quality of care [1]. Furthermore, this information provides insight into the patient’s perception of recovery and his/her adaptation to the chronic consequences [2, 32, 3].

The EQ-5D is a widely used generic HRQL instrument which has been validated for both description and valuation of quality of life impact [4, 5]. Its concise generic format makes it particularly useful for repeated measurements (minimal burden). However, the question whether dimensions should be added to the EQ-5D from a generic perspective has been debated and researched since its launch in the beginning of the 1990s. These additional dimensions are referred to as bolt-on dimensions (‘bolt-ons’); dimensions that describe additional specific health problems. Krabbe et al. [6] were the first to report that the extension of EQ-5D with a cognitive dimension adds information. Subsequently, several authors suggested valuable bolt-ons, including vision, energy, sleep, and skin irritation [7–9]. Two recent studies have used systematic approaches to identify possible bolt-ons for the EQ-5D from a range of items, including multiple items related to cognition and memory [10, 11]. Both these studies found that cognition is a relevant bolt-on for the EQ-5D, and possibly one of the most important ones [10, 11]. However, the relevance of the cognitive bolt-on in injury patients has not been investigated yet, even though cognitive impairments due to traumatic brain injury (TBI) and/or post-traumatic stress symptoms occur relatively frequently in this patient population [12–14].

Our paper addresses the following questions: (a) To what extent are the health impacts on the dimensions related, in particular: does cognition represent a statistically independent dimension?; (b) Do patients with cognitive problems report poorer EQ-VAS scores than patients without cognitive problems?; (c) What is the overall explanatory power of the EQ-5D-3L without and with the additional cognitive dimension using the EQ-VAS score as reference?

## Methods

### Study population and data collection

We analyzed data of the Dutch Injury Surveillance system (DISS). The DISS gathers data on intentional and unintentional injuries sustained by visitors to the emergency department (ED) of 13 hospitals throughout the Netherlands (12–15% coverage) [15]. The participating hospitals are a representative sample of hospitals in the Netherlands [16]. The ED visits recorded by these hospitals are generally considered to be representative for the total Dutch injury-related ED visits [16]. Information that is tracked by the DISS includes the cause, nature, and severity of injury, age, and sex of the patient and health care consumption during hospital admission (e.g., length of stay, admission to the Intensive Care unit). In the DISS hospitals, the ED patients are informed about the DISS registry with posters and leaflets that are placed in main patient areas of the ED. The posters and leaflets also explain that participants can withdraw from the DISS registry at any time.

#### DISS follow-up surveys

In 2001–2002 and 2007–2008, follow-up surveys were sent to a stratified sample of ED patients registered in the DISS 2.5 months after their visit to the ED due to an injury. The aim of these follow-up surveys was to collect data on HRQL, psychological consequences, return to work, and health care consumption after discharge from the hospital. These data are additional to the data tracked by the DISS. The 2001–2002 follow-up survey was sent to 10,612 patients and the 2007–2008 follow-up to 9907 patients. Severe and less common injuries were intentionally overrepresented for follow-up. For the follow-up surveys, a written informed consent form was sent by mail to the selected sample of ED patients together with the first follow-up questionnaire with the request to read, sign, and return with the filled-out questionnaire. In this way, written informed consent forms were obtained from all the respondents of the questionnaire. The follow-up studies were approved by the Medical Ethics Committee of the Academic Medical Center of Amsterdam (AMC).

Detailed information about the DISS follow-up surveys is presented in the articles by Polinder et al. [15] and Haagsma et al. [17].

### Data

#### EQ-5D outcome data

The EQ-5D is a generic HRQL instrument [5]. The instrument for self-assessment consists of a health classification (the EQ-5D descriptive system), and a subjective health rating (the EQ-VAS) with a score ranging from 0 (worst imaginable health state) to 100 (best imaginable health state). The EQ-5D descriptive system has five dimensions: mobility, self-care, usual activities, pain/discomfort, and anxiety/depression with ordinal response options. The EQ-5D is available in two response versions: the three-level version (EQ-5D-3L) and the more recent five-level version (EQ-5D-5L). The DISS follow-up surveys used the EQ-5D-3L. The ordinal levels of the EQ-5D-3L version are ‘no problems,’ ‘some problems,’ and ‘extreme problems/unable to.’ With five dimensions and three levels, the system creates 243 potential health profiles. A profile of ‘11111’ represents the best possible health state, whereas the profile ‘33333’ represents the worst possible health state. These health profiles have been valued by representative samples of the general population [18, 19]; from their values, a value set has been derived allowing the calculation of a utility score for any health profile. We used the value set derived from preferences of the general population of the Netherlands to calculate utility scores [20].

In current DISS follow-up surveys, the EQ-5D-3L was extended with a three-level cognition dimension covering aspects of memory, understanding, concentration, and thinking [6]. The text format was similar to that of the other dimensions, applying identical level descriptors. The verbatim presentation of the descriptor for the dimension added to the EQ-5D was as follows “Cognitive functioning, such as remembering, concentrating,” with the following levels (1) “I have no impairment of cognitive functioning,” (2) “I have some impairment of cognitive functioning,” (3) “I have severe impairment of cognitive functioning.” The EQ-5D-3L and the additional cognitive dimension (together labeled ‘EQ-5D + C’) were simultaneously administered. Only respondents with completed EQ-5D + C and EQ-VAS response were selected for analysis.

#### Supplemental DISS injury data

Apart from EQ-5D outcome data, the DISS follow-up surveys included questions on general socio-demographics, cause, and type of the injury according to the EUROCOST type of injury categories, health care use, expenditures, and return to work [21].

#### Socio-demographic data

The following socio-demographic data were available: gender, age in years, hospital admission after ED visit, comorbidity, and education level. Comorbidity was present if a patient reported one or more of the following health problems: chronic obstructive pulmonary disease (COPD) or asthma, heart disease including a previous myocardial infarction, previous stroke, diabetes mellitus, hernia, (rheumatoid) arthritis, and cancer. We coded the completion of higher professional education or university education as high education level.

### Data analysis

#### Dimension dependence

Two related questions are relevant if new dimensions are considered. (1) To what extent are the descriptive scores on the dimensions related (dependent)? (For example: the best level in mobility usually corresponds to best level in usual activities, or the level of self-care is never better than the lowest level of any of the other dimensions, a case of dominance or no relation beyond chance agreement.) (2) Are the contributions of the dimensions to the total utility score related? Note that two dimensions may be strongly related in a descriptive way, while they may still have independent roles in utility terms. Reversely, independent dimensions in a descriptive way may show strong interaction in utility terms, which may be cancelation or enhancement of the disutility associated with either of them. A new dimension at least should show both additional descriptive power and an independent utility role, preferably also at the non-extreme levels. This paper focuses on descriptive independence, question 1, and tentatively addresses question 2 by analysis of EQ-VAS data (see below). The EQ-VAS was used as a proxy of HRQL in this analysis.

To check descriptive dependence, we created cross tables between pairs of dimensions, specifically checking instances of dominance/subordination of the cognitive dimension. The following procedure was developed. We considered the profiles with level 3 (L3; extreme problems) on one dimension (A) and level 1 (L1; no problems) on another dimension (B). Then we defined ‘dominance of dimension A across B’ as the presence of *less* combinations of A-L3 and B-L1, than of A-L1 and B-L3, corrected for chance frequency of these combinations. This definition catches ‘negative’ dominance, defined as the mechanism that dysfunction in dimension A is paralleled by dysfunction in each other dimension, limiting the probability of a high level. In our context ‘positive’ dominance is of no interest, i.e., a high level of dimension A limits the presence of poor levels elsewhere. The authors formulated hypotheses regarding the dominance of domains through discussion. For the cognition domain, we hypothesized that cognition dominates self-care and usual activities. It was deemed plausible that severe cognitive problems co-occur with severe problems with self-care and usual activities, but not the other way around. The plausibility of the remaining domains combination falls outside the scope of this paper, and are therefore not further discussed.

In our data analysis, we first estimated the probability of all possible level 3 and level 1 dimension combinations under independence, based on multiplication of the marginal frequencies of the levels per dimension using the following formula:

$${\text{Probability (D1}}\_{\text{L1}}|{\text{ D2}}\_{\text{L3)}}\,=\,{\text{prevalence (D1}}\_{\text{L1)}} \times {\text{prevalence (D2}}\_{\text{L3)}},$$

where D1_L1 is dimension 1 (e.g., mobility) level 1 and D2_L3 is dimension 2 (e.g., self-care) level 3.

For instance, if mobility_L3 had a prevalence of 10% and usual activities_L1 had a prevalence of 60%, the estimated expected conditional probability of mobility_L3 and usual activities_L1 is 6%. Then we listed all prevalent EQ-5D + C profiles of our dataset, and selected among them the pairs with contrasting results (some pairs qualify for multiple contrasts, e.g., the profile 112,313 contains 6 L1–L3 contrasts). We compared the number of L1–L3 versus L1–L3 contrasts for dimension combinations with cognition as one of the two dimensions, and calculated the relative frequency of both contrasts, i.e., the observed frequencies relative to chance frequencies. E.g., cognition level 3 and pain/discomfort level 1 versus pain/discomfort level 3 and cognition level 1. The ratio of the relative frequencies (cognition L1 & dimension × L3 as denominator) decides on dominance: if it is 1.0 then dimensions are independent, if it is < 1.0 than cognition dominates, if > 1.0 then cognition is subordinate.

Dimension dependency was additionally investigated by calculating Spearman’s rank correlation coefficients between the six EQ-5D + C dimensions.

#### Effect of cognition level on EQ-VAS

The effect of cognition level on the EQ-VAS was investigated by calculating the average EQ-VAS score for each cognition level irrespective of the corresponding EQ-5D profile. The same step was repeated for EQ-5D profiles with relatively severe and mild problems on the other dimensions, which allowed for direct comparison of the EQ-VAS scores as only the cognition dimension levels differed within the complete EQ-5D + C profiles.

#### Explanatory power analysis of all dimensions

We then predicted the EQ-VAS score from the EQ-5D dimensions, the cognitive dimension, and the socio-demographic factors. Univariate and multivariate regression analysis was applied. All descriptive EQ-5D + C dimensions were dummy coded (= standard): ‘some problems’ and ‘severe problems’ with ‘no problems’ as reference category. Separate and combined analyses were performed for participants with reported full health (EQ-5D profile of 11111) and without full health because of the combined effect of many respondents reporting to be in full health and the non-linear relations in the upper part of the scale. Only complete responses of the EQ-5D, cognition question, and EQ-VAS were selected for analysis.

Firstly, we performed univariate regression analyses and predicted the EQ-VAS with the EQ-5D and cognition attributes. Subsequently, a multivariate regression analyses model was constructed including the original EQ-5D attributes. In the second step, the cognition attributes were added to the model in order to examine the additive effect. Multivariate regression analysis was also used to assess the explanatory power of any set of combinations of five of the six EQ-5D + C dimensions.

Secondly, the EQ-5D attributes, cognition attributes, and all socio-demographic characteristics were simultaneously offered (forced entry) to a multivariate regression model explaining the EQ-VAS score. The initial model contained first-degree interactions. The backward deletion strategy was employed, starting from a model with 16 variables. We deleted non-significant predictors from the model until only significant predictors remained (*p* < 0.05). The regression analyses were repeated for patients with specific injury categories to explore the effect of the cognition dimension among patients with and without traumatic brain injury (clinical known-group comparison).

All analyses were conducted using SPSS V.24 (Statistical Package for Social Sciences, Chicago, Illinois, USA).

The following hypotheses were formulated:

1. There are no redundancies and dependency patterns in the domains of the EQ-5D-3L, but they may exist when cognition domain is added.
2. There is no added descriptive value of cognitive information, i.e., the coefficient size of cognitive information (in cross-sectional explanatory analysis) is smaller than that of the least important EQ-5D3L domain, regardless whether socio-demographics are added.
3. The explanatory power of the EQ-5D + C is higher compared to the EQ-5D in TBI patients due to specific cognitive symptoms after TBI.

## Results

### Descriptive results

**Fig. 1:**
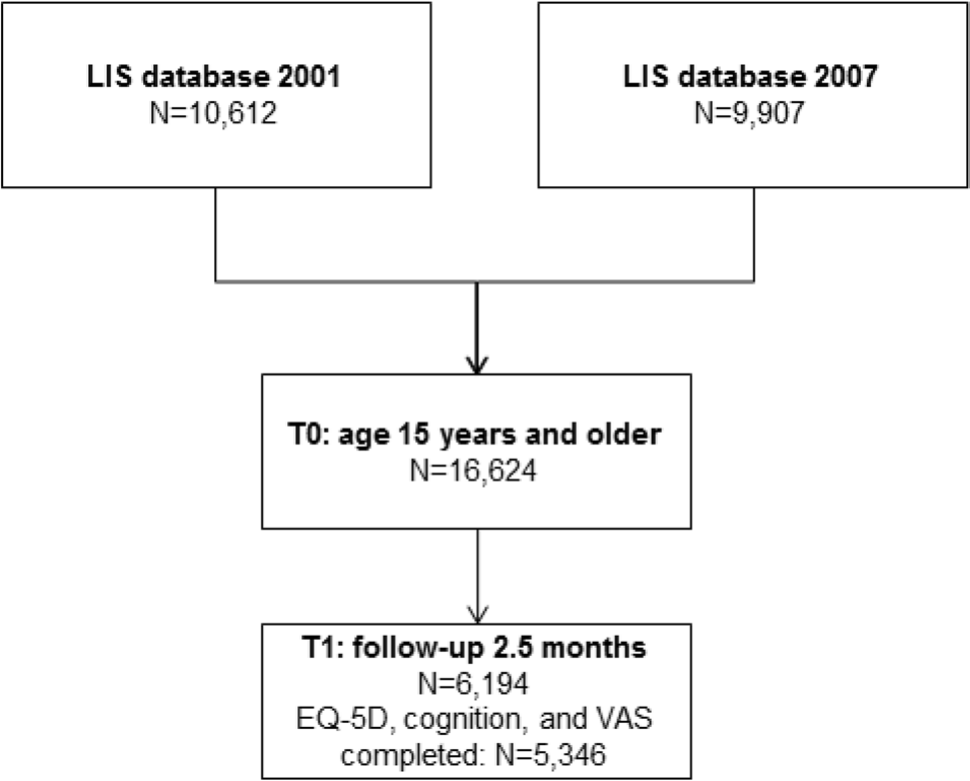
Patient flow chart

Figure 1 describes the flow chart of the participant selection, follow-up, and response. The combined response rate of the 2001 and 2007 follow-up surveys was 6194 (37.3%), of which 5346 (32.2%) had complete responses of the EQ-5D + C and EQ-VAS at 2.5 months follow-up. The characteristics of the respondents are listed in Table 1. Responders were significantly older than non-responders (median age 49.9 vs. median age 47.5, *p* < 0.01); the proportion females was higher (50.7% vs. 43.4%, *p* < 0.01); and the proportion of patients admitted to hospital was higher (56.1% vs. 46.9%, *p* < 0.01).

**Table 1:** Characteristics of the respondents (aged 15 and older)

Respondent demographics	
n	5346
Age (mean)	49.9 (SD 21.5)
Females	50.7%
Type of injury	
Skull-brain injury	11.8%
Facial fracture, eye injury	3.9%
Spine, vertebrae	5.0%
Internal organ injury	6.1%
Upper extremity fracture	12.9%
Upper extremity, other	6.4%
Hip fracture	6.8%
Lower extremity fracture	16.1%
Lower extremity, other	9.9%
Superficial injury, open wounds	16.4%
Burns	0.8%
Poisonings	0.8%
Other injury	3.3%
Hospitalization	56.1%
Length of stay^a^ (median, IQR)	4.0 [2.0–11.0]

*SD* standard deviation, *IQR* interquartile range

^a^Median length of stay of patients who were admitted to hospital after their ED visit

In total, 150 out of 243 (61.7%) possible EQ-5D profiles were reported. The responses to the dimensions of the EQ-5D + C indicated that the pain/discomfort dimension was most affected at 2.5 months follow-up (62.4% reporting any problems), followed by usual activities (57.6% reporting any problems). The cognitive dimension was the least affected (19.6% reporting any problems). Extreme problems (level 3) were most frequently reported in the usual activities dimension (15.5%), whereas extreme problems were least reported in the mobility and cognition dimensions (both 2.7%). A total of 1419 respondents (26.5%) reported no problems on any dimension and thus had an EQ-5D profile of 11111. Respondents with a 11111 profile were more likely to be male (61.8% vs. 44.8%; *p* < 0.01) and younger (mean age 41.8 years vs. 52.9 years; *p* < 0.01), less likely to be admitted to the hospital (30.9% vs. 65.2%, *p* < 0.01) and had fewer comorbidities (16.8% vs. 43.8%, *p* < 0.01).

#### Dimension dependency

**Fig. 2:**
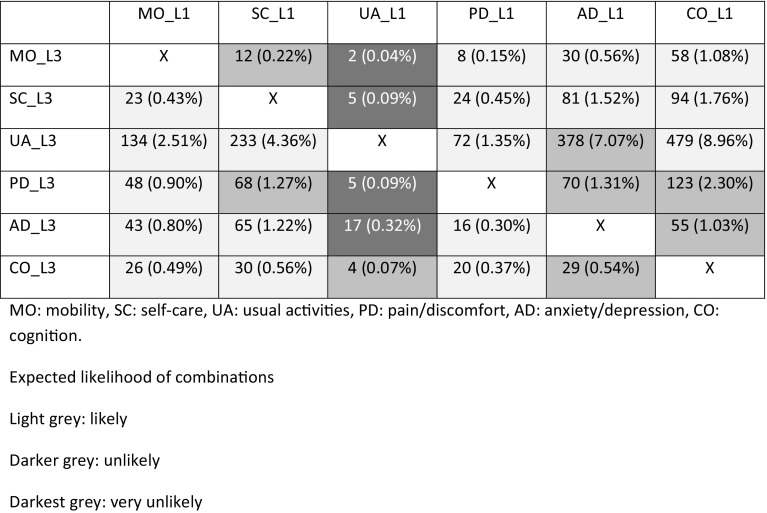
EQ-5D + C level 1 and 3 combinations, in absolute numbers and expressed as the percentage of total EQ-5D + C profiles (*n* = 5346)

Figure 2 describes our expectations regarding the likelihood of possible contrasting dimensions. We distinguished likely, unlikely, and very unlikely contrasting dimension levels which were based on agreement between the authors. We defined ‘unlikely’ as unlikely, but possible in certain cases. Our results showed that all unlikely combinations did occur (0.04–1.31% of all observed combinations). Especially, the cognition level 1 and pain/discomfort level 3 was observed relatively frequent (n = 132, 2.3% of total observed EQ-5D + C profiles) (Fig. 2). We found that level 3 cognition in combination with level 1 combinations on all other dimensions were uncommon (< 1%), especially with usual activities. On the contrary, level 1 cognition and level 3 of other dimension combinations occurred more frequently (> 1%), especially usual activities level 3 (9.0%). The ratio of the relative frequencies of cognition L3 & dimension L1 and cognition L1 & dimension L3 was < 1.0 for all the dimensions. These findings indicate that cognition is dominant over the other dimensions: extreme cognitive problems and no problems on other dimensions are uncommon, whereas extreme problems on other dimensions frequently occur with no cognitive problems. The Spearman’s rank correlation coefficients between the six dimensions ranged from 0.592 (usual activities and pain/other) to 0.223 (mobility and cognition). The Spearman’s correlation coefficients between cognition and each of the EQ-5D dimensions was lower than 0.31, except for the dimension anxiety/depression (Spearman’s correlation coefficient = 0.42). For the other dimensions, only the Spearman’s correlation coefficient for mobility and anxiety/depression was lower than 0.31 (Spearman’s correlation coefficient = 0.30).

#### EQ-VAS by cognition level for various profiles

The average EQ-VAS for each cognition level, combining all profiles given a particular cognition level, is described in Table 2. The EQ-VAS significantly decreased when cognitive problems increased [no problems mean EQ-VAS 74.5 (95% confidence interval (CI) 74.0–75.0)]; moderate problems EQ-VAS 57.7 (95% CI 56.5–59.0); extreme problems mean EQ-VAS 41.1 (95% CI 37.7–44.4), which showed that cognitive problems are associated with a decrease of the EQ-VAS. The analyses for specific profiles (Table 2) show the same trend, although the decrease of the EQ-VAS was not significant for all profiles, probably due to low sample sizes.

**Table 2:** EQ-VAS score by cognition level for overall EQ-5D profiles and for exemplary EQ-5D profiles with the most frequently reported level 3 cognition

EQ-5D profile	Cognition level					
	1		2		3	
	*n*	Mean (95% CI)	*n*	Mean (95% CI)	*n*	Mean (95% CI)
All	4298	74.5 (74.0–75.0)	949	57.7 (56.5–59.0)	157	41.1 (37.7–44.4)
11111	1365	85.7 (85.1–85.4)	52	79.9 (76.6–83.2)	2	52.2 (47.6–57.4)
21221	444	70.5 (69.2–71.9)	51	61.6 (57.1–66.0)	2	47.5 (23.0–72.0)
22221	176	63.2 (60.8–65.6)	37	61.2 (56.6–65.8)	2	40.0 (20.4–59.6)
23322	13	46.9 (38.4–55.4)	17	45.7 (39.3–52.2)	15	39.1 (31.4–46.8)
33322	7	40.0 (25.7–54.3)	16	39.2 (33.6–44.8)	9	32.2 (24.5–39.9)
33333	3	18.3 (15.1–21.6)	6	17.5 (1.5–33.5)	10	14.0 (6.3–21.7)

*95% CI* 95% confidence interval

#### Univariate regression analysis

Univariate regression analyses indicated that all EQ-5D + C dimensions were significantly associated with the EQ-VAS (Table 3). The direction of the relative size of level 2 and level 3 impairments of the same dimension was as expected, with extreme problems on any dimension resulting in greater deficits of the EQ-VAS compared to moderate problems. In univariate analysis, extreme problems with performing usual activities explained most of the variance of the EQ-VAS (17.3%). Moderate and extreme cognitive problems explained, respectively, 8.7% and 6.2% of the variance of the EQ-VAS. For the non-11111 profiles, moderate and extreme cognitive problems explained both 6.1% of the variance of the EQ-VAS.

**Table 3:** Association between the EQ-VAS and the EQ-5D and cognition dimensions (univariate analyses)

EQ-5D dimension	*R* ^2^	Unstandardized *b* (95% CI)	*p* value
Mobility level 2	0.128	− 14.5 (− 15.5 to − 13.5)	< 0.001
Mobility level 3	0.082	− 35.1 (− 38.3 to − 32.0)	< 0.001
Self-care level 2	0.104	− 16.0 (− 17.2 to − 14.7)	< 0.001
Self-care level 3	0.110	− 30.1 (− 32.3 to − 27.8)	< 0.001
Usual activities level 2	0.035	− 7.5 (− 8.6 to − 6.5)	< 0.001
Usual activities level 3	0.173	− 22.9 (− 24.2 to − 21.5)	< 0.001
Pain/discomfort level 2	0.079	− 11.3 (− 12.3 to − 10.3)	< 0.001
Pain/discomfort level 3	0.104	− 30.3 (− 32.7 to − 27.9)	< 0.001
Anxiety/depression level 2	0.126	− 17.1 (− 18.3 to − 15.8)	< 0.001
Anxiety/depression level 3	0.076	− 30.0 (− 32.8 to − 27.2)	< 0.001
Cognition level 2	0.087	− 15.7 (− 17.1 to − 14.3)	< 0.001
Cognition level 3	0.062	− 30.5 (− 33.7 to − 27.3)	< 0.001

*95% CI* 95% confidence interval

For profile 11111 respondents cognition level 2 explained, respectively, 0.8% of the variance of the EQ-VAS. For cognition level 3, this could not be calculated, since there were only two respondents that reported a 11111 profile and extreme cognitive problems.

### Multivariate regression analysis

The additional cognitive dimension increased the explanatory power of the multivariate model from 45.6% (EQ-5D) to 46.9% (EQ-5D + C). Exploratory analyses showed that using any set of five of the six EQ-5D + C dimensions resulted in almost identical explained variance, and adding the remaining 6th dimension resulted in a similar additional impact (Table 4).

**Table 4:** Explanatory power of multivariate models that included any set of the EQ-5D and cognition dimensions (multivariate analyses)

Selection of EQ-5D + C dimensions	*R* ^2^	*F* value	*p* value
MO + SC + UA + PD + AD	0.456	448.1	< 0.01
MO + SC + UA + PD + AD + CO	0.469	393.2	< 0.01
MO + UA + PD + AD + CO	0.459	453.3	< 0.01
MO + SC + PD + AD + CO	0.456	447.3	< 0.01
MO + SC + UA + AD + CO	0.448	433.1	< 0.01
MO + SC + UA + PD + CO	0.445	427.4	< 0.01
SC + UA + PD + AD + CO	0.454	443.7	< 0.01

*MO* mobility, *SC* self-care, *UA* usual activities, *PO* pain/other, *AD* anxiety/depression, *CO* cognition

The final model, which explained 48.7% of the variance of the EQ-VAS, included the EQ-5D and cognition attributes and comorbidity. According to the model, having comorbid disease is associated with a significant decrease of the EQ-VAS (Table 5).

**Table 5:** Explanatory power of the multivariate model that included the EQ-5D + C dimensions and comorbidity

EQ-5D dimension	Unstandardized b	*p* value
		< 0.01
Constant	87.4	< 0.01
Comorbidity	− 4.0	< 0.01
Mobility level 2	− 4.8	< 0.01
Mobility level 3	− 16.4	< 0.01
Self-care level 2	− 4.2	< 0.01
Self-care level 3	− 7.2	< 0.01
Usual activities level 2	− 5.6	< 0.01
Usual activities level 3	− 10.1	< 0.01
Pain/discomfort level 2	− 5.3	< 0.01
Pain/discomfort level 3	− 16.1	< 0.01
Anxiety/depression level 2	− 7.4	< 0.01
Anxiety/depression level 3	− 11.6	< 0.01
Cognition level 2	− 6.3	< 0.01
Cognition level 3	− 10.9	< 0.01
*F* value	192.6	
Corresponding *p* value	*p* < 0.01	
*R* ^2^	0.487	

### Traumatic brain injury versus other injury

For patients with traumatic brain injury, the additional cognition dimension increased the explanatory power of the multivariate model from 55.6% (EQ-5D) to 56.5% (EQ-5D + C). For non-TBI patients, this increase was slightly larger, namely, from 44.5% (EQ-5D) to 45.8% (EQ-5D + C).

## Discussion

Our results showed that extreme cognitive problems and no problems on other dimensions are uncommon, whereas extreme problems on other dimensions frequently occur with no cognitive problems. Moreover, we found that the decrease of HRQL measured with the EQ-VAS resulting from cognitive problems was significant. These findings indicate that cognition is dominant over the other dimensions. The additional cognitive dimension increased the explanatory power of the multivariate EQ-5D-3L attributes model from 45.6% (EQ-5D) to 46.9% (EQ-5D + C). This increase is small but similar to adding one of the original EQ-5D dimensions to any set of five EQ-5D + C dimensions.

The performance of the cognition bolt-on has been investigated in previous studies. Krabbe et al. [6] compared valuations (by means of the EQ-VAS) elicited from EQ-5D + C descriptions with parallel EQ-5D descriptions in members of Dutch university staff members. The content validity of the EQ-5D improved by adding cognition. The authors emphasized the importance of considering the inclusion of a cognitive dimension. The employed methods were too crude to quantify the increased explanatory power, however. Wolfs et al. [22] investigated the construct validity and responsiveness of the EQ-5D + C and the EQ-5D in older adults with cognitive impairments using the Mini Mental State Examination (MMSE) as reference. Regarding construct validity, similar correlations between the EQ-5D and the MMSE and between the EQ-5D + C and the MMSE were found, which indicated that there were no differences in construct validity. The EQ-5D and the EQ-5D + C were both responsive to changes in the MMSE, but the EQ-5D performed slightly better. The study of Wolfs et al. [22] is difficult to compare to the results of our study, as the MMSE was used as reference and the increased explanatory power was not investigated.

There does not seem to be consensus as to what increase in R-square is actually meaningful [7]. For example, Swinburn et al. [23] reported that the addition of skin irritation and self-confidence dimensions to the EQ-5D-3L increased the R-square with 22% for psoriasis patients and concluded that the addition of dimensions was much better at predicting outcomes. Whynes [24] reported that the addition of five dimensions increased the explanatory power up to 56%, which was defined a substantial improvement. However, Yang et al. [9] reported that an increase in explanatory power (TTO outcome) of 6% as a result of adding a sleep dimension was not a significant improvement. Compared to these results, an increase in explanatory power of 1.3% is relatively low. However, it is comparable to increase in explanatory power by other EQ-5D dimensions. Moreover, the explanatory power of the multivariate model that included all EQ-5D dimensions was 45.6%. This is in the same order of magnitude as the explanatory power of a multivariate regression model that included the EQ-5D-3L dimensions in community samples [7, 25]. This indicates that variation in HRQL as measured with the EQ-VAS is affected by aspects that are not covered by the current EQ-5D items. This is underlined by the variation in EQ-VAS scores of respondents with a 11111 profile.

### Strengths and limitations

The most important strength of our study is the large number of participants, which allowed for analyses of subgroups, including the additional effect of the cognition bolt-on for specific injury types, without losing statistical power. Furthermore, EQ-5D + C responses were consistent, meaning that extreme problems on any dimension resulted in greater deficits of the EQ-VAS compared to some problems. A limitation of our study is that the follow-up survey was administered 2.5 months post-injury. Many injured patients recover sooner than 2.5 months [15], especially patients with minor injuries such as open wounds, superficial injuries, and contusions. This applies also to mild traumatic brain injury such as concussions in particular, as it is known that cognitive problems are most present within 2 weeks post-injury [26]. Second, a limitation of our study was that the respondents were not a representative sample of the patients who are registered in DISS. Firstly, severe and less common injuries were intentionally overrepresented for follow-up. Secondly, young males were less likely to respond to our survey. As a result, the respondents were on average older and the percentage of females was higher compared to the patients that were registered in the DISS.

A third limitation of this study is that the EQ-VAS was used as a proxy of HRQL. A well-known problem of the EQ-VAS is that it is subject end of scale bias, a measurement bias that causes respondents to avoid the extremes of the scale.

In this study, we administered the cognitive dimension question immediately after the original EQ-5D and before the EQ-VAS question on the same page. We assumed that participants answered the original EQ-5D questions in the same way regardless of whether the cognition question was included. However, the responses to the original EQ-5D questions may have been influenced by the added cognition questions and the sequence of questions.

### Recommendations for future research

For future research, we recommend to investigate the added value of the cognitive bolt-on in a sample of trauma patients shortly after sustaining their injury, e.g., maximally 2 weeks post-injury to ensure that patients are still experiencing symptoms. We also recommend investigating the added value of the cognitive dimension in other patient groups, such as patients suffering from permanent traumatic brain injury, but also in patients groups that have illnesses that are not related to cognitive impairments. We furthermore recommend to investigate the effects of adding a cognitive dimension to the EQ-5D-5L. The EQ-5D-5L improved the measurement properties and discriminatory power in comparison with the EQ-5D-3L among different patient groups [27]. Therefore, it is possible that a cognition bolt-on explains more variance of the EQ-VAS when the EQ-5D-5L is used.

## Conclusion

The addition of the cognitive dimension increased the explanatory power of the EQ-5D-3L. Although the increase in explanatory power was relatively small after the cognition dimension was added, the decrease of HRQL (measured with the EQ-VAS) resulting from cognitive problems was comparable to the decreases resulting from other EQ-5D dimensions.
